# Unusual Lipid Components of *Legionella gormanii* Membranes

**DOI:** 10.3390/metabo12050418

**Published:** 2022-05-06

**Authors:** Elżbieta Chmiel, Christina E. Galuska, Piotr Koper, Bożena Kowalczyk, Teresa Urbanik-Sypniewska, Marta Palusińska-Szysz, Beate Fuchs

**Affiliations:** 1 Department of Genetics and Microbiology, Institute of Biological Science, Faculty of Biology and Biotechnology, Maria Curie-Skłodowska University, Akademicka 19, 20-033 Lublin, Poland; e.chmiel@sp27.lublin.eu (E.C.); piotr.koper@mail.umcs.pl (P.K.); b.kowalczyk746@wp.pl (B.K.); teresa.urbanik-sypniewska@poczta.umcs.lublin.pl (T.U.-S.); 2 Core Facility Metabolomics, Research Institute for Farm Animal Biology (FBN), Wilhelm-Stahl-Allee 2, 18196 Dummerstorf, Germany; galuska.christina@fbn-dummerstorf.de

**Keywords:** *Legionella gormanii*, phosphatidylcholine, ceramides, lipidomic analysis

## Abstract

*Legionella* spp. cause Legionnaires’ disease with pneumonia as the predominant clinical symptom. *L. gormanii* is the second most prevalent causative agent of community-acquired pneumonia after *L. pneumophila*. The study aimed to characterize the lipidome of *L. gormanii* membranes and the importance of these analyses in bacterial chemotaxonomy. Lipidomic analyses based on ultra-high performance liquid chromatography-mass spectrometry allowed the detection of individual molecular species of a wide range of *L. gormanii* membrane lipids contained in the outer (OM) and inner membranes (IM). The lipid profile comprised glycerolipids (triglycerides, diglycerides), phospholipids (phosphatidylcholine, phosphatidylethanolamine, phosphatidylglycerol, cardiolipin), and sphingolipids (ceramides, hexosylceramides). The most abundant lipid fraction in the IM and OM were phospholipids. The lipidomic analysis showed that two independent phosphatidylcholine (PC) synthesis pathways operating in *L. gormanii*: the PE-methylation (PmtA) pathway and the PC synthase (Pcs) pathway. Comparison of the molecular profile of PC species contained in the lipids of *L. gormanii* membranes cultured on the medium, with and without exogenous choline, showed quantitative differences in the PC pool. An unusual feature of the *L. gormanii* lipids was the presence of ceramides and hexosylceramides, which are typical components of eukaryotic cells and a very small group of bacteria. To the best of our knowledge, this is the first report of the occurrence of ceramides in *Legionella* bacteria.

## 1. Introduction

Legionellosis is the common name for disease syndromes caused by exposure to *Legionella* bacteria. The disease varies in severity from a mild febrile illness (Pontiac fever) to a serious and fatal form of pneumonia (Legionnaires’ disease, LD). *Legionella* species are environmental Gram-negative bacteria with the ability to survive and replicate within protozoan hosts. This adaptation of the bacteria to the intracellular niche in nature leads to invasion and growth within human alveolar macrophages [1]. Among the 69 *Legionella* spp. described, *L. pneumophila* is the leading cause of LD, and *L. pneumophila* serogroup 1 is associated with almost 85–90% of the cases worldwide [2]. However, non-pneumophila species of *Legionella* are also important human pathogens that pose a particular risk to immunocompromised and splenectomized individuals [3].

*L. gormanii* is one of the 28 *Legionella* species that have been associated with human disease. It was first isolated clinically from a 64-year-old pneumonia-affected woman with systematic lupus erythematosus and adenocarcinoma [4]. Although *Legionella* species are rarely isolated from children, *L. gormanii* has been found in this patient group [5,6]. In the process of lung tissue invasion and colonization, lipases and phospholipases are important virulence factors of *Legionella* spp., as they degrade the surfactant covering small airways, bronchioles, and the alveolar surface. The lung surfactant is composed of approximately 10% of protein and 90% of lipids. Approximately 80% of these lipids are accounted by phosphatidylcholine (PC), which represents a good target for bacterial phospholipase A (PlaB) in the destruction of this important layer, thus contributing to impairment of lung function. *L. gormanii* showed cell-associated phospholipase A and lysophospholipase A activities in quantities comparable to *L. pneumophila* [7].

The cell envelope of Gram-negative bacteria consists of two distinct layers, the outer (OM) and the inner (IM) membranes, separated by the periplasm with a thin layer of peptidoglycan. The inner membrane contains of phospholipids covering the inner and the outer leaflets. The OM is a highly asymmetric lipid bilayer, comprising an inner leaflet enriched in phospholipids and an outer leaflet containing lipopolysaccharide (LPS). This strict OM asymmetry is important for the proper functioning of the bacterial cell, as it constitutes a permeability barrier, protects against the host’s immune system and prevents the penetration of toxic compounds into the cell. Additionally, the *Legionella* OM mediates direct contact with other organisms, determining highly specific interactions with the host cell. 

Unlike in the Gram-negative model bacterium *Escherichia coli* and the Gram-positive *Bacillus subtilis*, but similar to eukaryotic cells, *Legionella* membranes are rich in PC and this phospholipid is necessary for the full virulence of these pathogens. *L. pneumophila* mutant defective in PC synthesis showed a poorly functioning type IV secretion system (Dot/Icm), which delivers effectors required for intracellular multiplication in the cytosol of infected host cells [8]. Synthesis of PC in *Legionella* cells occurs via two independent biosynthesis pathways, i.e., the phosphatidylethanolamine (PE) methylation pathway and the PC synthase (Pcs) pathway. In the methylation pathway, PE is methylated three times to form PC via phospholipid *N*-methyltransferase (PMT). This enzyme is a small cytosolic protein encoded by the *pmtA* gene. In the Pcs pathway, choline is condensed directly with CDP-diacylglyceride to produce PC in a reaction catalyzed by the phosphatidylcholine synthase (Pcs), which occurs exclusively in bacteria [9]. The synthase is a highly hydrophobic protein containing up to eight transmembrane helices with *N*- and *C*-termini located inside the bacterial cell. A comparative analysis of the nucleotide sequences of *pcs* showed that these genes share a high sequence identity among *Legionella* spp.; therefore, they can be used to identify this bacterial group [10]. Lipids of *L. gormanii* were estimated to account for 4% of dry weight, with PE (50%) and PC (26%) representing the major glycerophospholipid classes. Other phospholipids that build the membranes of the bacterium were cardiolipin (CL, 21%) and phosphatidylglycerol (PG, 9%). Similar to another *Legionella* spp., *L. gormanii.* utilizes exogenous choline in the Pcs pathway to synthesize PC. These bacteria cultured on the medium with choline synthesized by 21% more PC and 12% PE and by 9% lower CL levels, compared with the bacteria grown on a medium without the addition of choline [10]. The supplementation of the growth medium with choline resulted in a higher concentration of proteins in *L. gormanii*, which indicated that the phospholipid environment affected the assembly of membrane proteins [10]. The synthesis of PC from an exogenous precursor influences the phospholipid and protein *Legionella* components and the interactions between these components lead to changes in sensitivity to antimicrobial protein and peptides. Our previous study showed that *L. dumoffii* cultured in the presence of choline was more sensitive to the antimicrobial peptide defensin and apolipophorin III (apoLp-III) isolated from *Galleria mellonella* hemolymph [11]. ApoLp-III, i.e., an insect homolog of human apolipoprotein E exerted a bactericidal effect on *L. gormanii* cells at an 8-fold lower concentration, compared to *L. dumoffii*, possibly reflecting differences in the cell surface properties [12]. 

In this study, the remarkable lipid components of *L. gormanii* membranes are reported and this strain is, therefore, a novel representative of a narrow group of bacteria containing phosphatidylcholine and ceramides. 

## 2. Results

### 2.1. Composition of Fatty Acids of Individual Classes of Phospholipids

The fatty acid compositions of phosphatidylethanolamine (PE), phosphatidylcholine (PC), dimethylphosphatidylethanolamine (dMePE), phosphtidylglycerol (PG) and cardiolipin (CL) were determined. Each of these phospholipids showed a distinctive and characteristic fatty acid pattern (Table 1).

In the PE class, saturated, branched (mainly i16:0 (22%) and a15:0 (19%)) acids and a smaller amount of a17:0 (7%) acid constituted approximately half of total FAs. This fraction was also characterized by a high content of cyclopropyl 17:0 (18%) and straight-chain 16:0 (17%) acid. The FA composition of the dMePE class included considerable quantities of octadecanoic and hexadecanoic acids, which, together, accounted for 65%. This phospholipid also contained a substantial amount of cyclopropyl 17:0 acid (19%) and small quantities of unsaturated 18:1∆^9^, as well as long-chain FAs (22:0, 1.5%; 24:0, 2%).

The PC fraction was predominantly composed of cyclopropyl 17:0 acid, which accounted for 22% of the total FAs of this phospholipid. Both branched (*iso* and *anteiso*) and unbranched FAs were determined in this class. Hexadecanoic (18%) and octadecanoic (16%) acids with straight-chain saturated FAs were the most abundant. Branched FAs accounted for 30% of all acids, with a dominance of i16:0 (12%) and a15:0 (8%). 

The PG class was characterized by high content of methyl-branched FAs (a15:0, 17%; i16:0, 19%; a17:0, 9%). 

Compared to other phospholipid classes, the CL class contained the largest amount of unsaturated (16:1∆^9^, 6%; 18:1∆^9^, 5%) acids, as well as long-chain FAs with the number of carbon atoms ranging from 20 to 24.

### 2.2. Separation of the Inner (IM) and Outer (OM) Membranes

**Figure 1:**
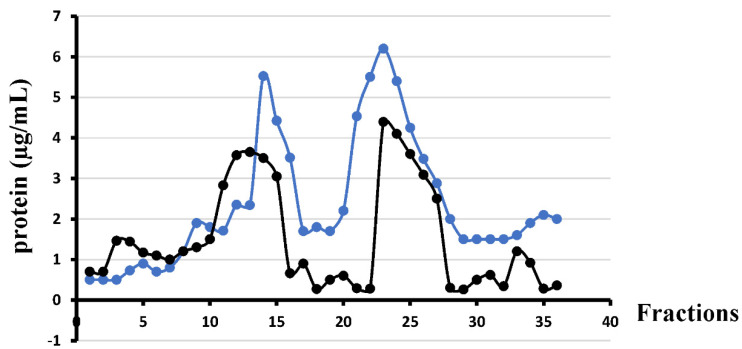
Separation of the *L. gormanii* inner (IM) and outer membrane (OM) by sucrose density gradient centrifugation. The 1 mL fractions were collected from the top of the gradient and assayed for the presence of protein (µg/mL). Black line: bacteria cultured on the exogenous choline non-supplemented medium; blue line: bacteria cultured on the exogenous choline-supplemented medium.

To determine the lipid profile in the membranes of *L. gormanii* cultured on the medium with and without exogenous choline, the bacteria were separated into IM and OM fractions by sucrose density gradient ultracentrifugation. Biochemical analyses confirmed the effectiveness of membrane separation. Measurement of the protein concentration in each fraction showed two main peaks, one corresponding to fractions 13–17 and 10–16 for bacteria grown with and without the addition of exogenous choline, respectively, and the second peak corresponding to fractions 20–28 and 22–28 for bacteria cultured with and without choline supplementation, respectively (Figure 1). NADH oxidase activity was concentrated in pooled fractions (12–16 for the choline-supplemented bacteria and 10–15 for the choline non-supplemented bacteria) corresponding to the IM. The activity of NADH oxidase was 171 μmol min^−1^ mL^−1^ and 239 μmol min^−1^ mL^−1^ for bacteria cultured with exogenous choline and on the standard medium, respectively. 

### 2.3. Lipidomic Analysis

**Figure 2:**
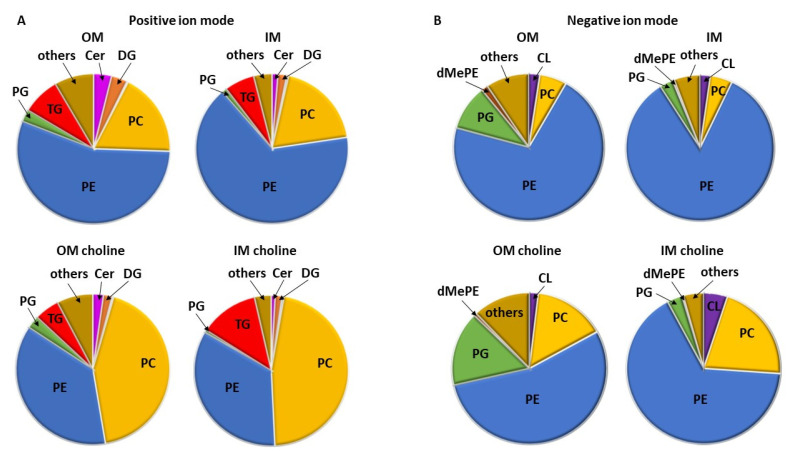
Lipid classes in the OM and IM of *L. gormanii* grown on a medium with and without the addition of exogenous choline. The lipid classes were analyzed by LC-MS in the positive (**A**) and negative ion mode (**B**).

Lipidomic analyses based on the UHP LC-MS/MS method allowed detection and relative quantification of individual molecular species of a wide range of *L. gormanii* membrane lipids contained in the OM and IM. The lipid profile comprised glycerolipids (triglyceride, TG; diglyceride, DG), phospholipids (phosphatidylcholine, PC; phosphatidylethanolamine, PE; phosphatidylglycerol, PG; cardiolipin, CL), and sphingolipids (ceramides, Cer, hexosylceramides, Hex1Cer). The most abundant lipid fraction in the IM and OM were phospholipids (Figure 2).

### 2.4. Phospholipid Profile

**Figure 3:**
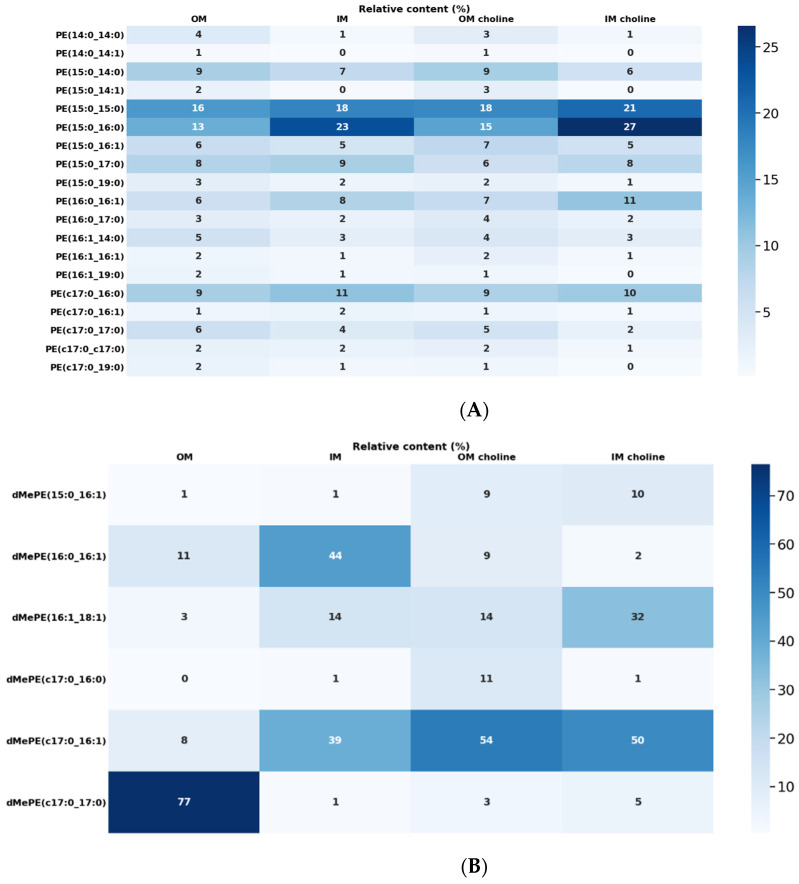

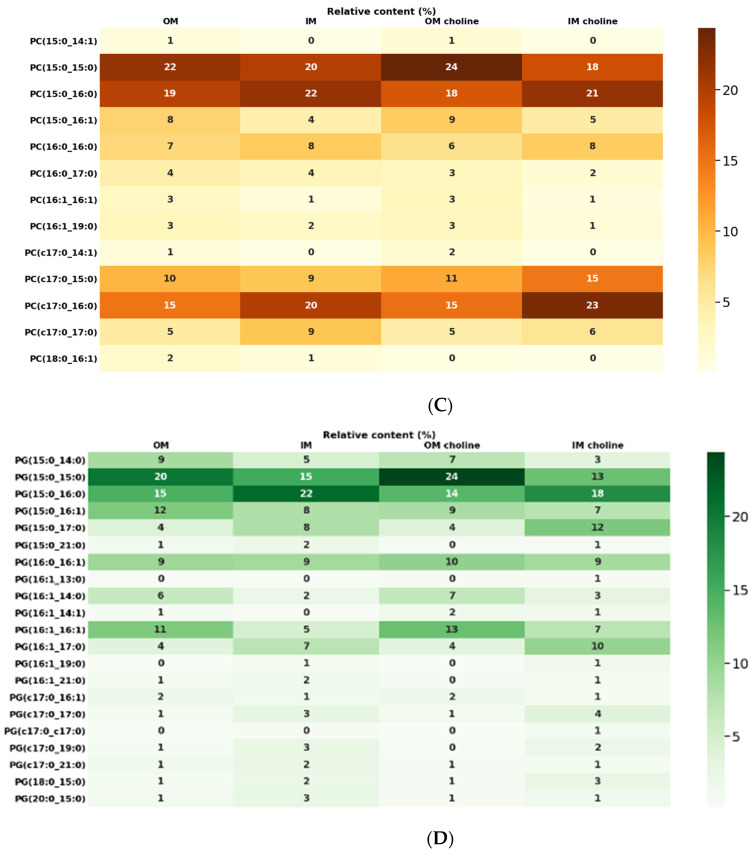
Heatmap displaying the relative abundances of PE (**A**), dMePE (**B**), PC (**C**), and PG (**D**) molecular species in total lipid extracts of the OM and IM of *L. gormanii* cultured on the standard medium and with exogenous choline. PE, PC and PG were analyzed by LC-MS(/MS) in the positive ion mode. The phospholipid class dMePE was analysed by LC-MS(/MS) in the negative ion mode.

Among polar lipids, PE was the dominant class in the OM and the IM. The analysis of the molecular profile of PE showed the presence of 35 PE species. The main species were shown in Figure 3A. The most abundant species were PE15:0_15:0 (16%) in the OM and IM (18%), PE15:0_16:0 (13% and 23% in the OM and IM, respectively), and PEcyclopropyl17:0_16:0 (9%) in the OM and (11%) IM (Figure 3A). Spearman’s rank correlation coefficient for PE species content between the membranes was positive but moderate at 0.67 indicating that the PE compositions are characteristic for the IM and OM.

Methylated derivatives of PE were identified as well. The profile of dMePEs showed that the most abundant species were dMePE cyclopropyl17:0_17:0 (77%), dMePE16:0/16:1 (11%) in the OM and dMePE16:0_16:1 (44%), dMePE cyclopropyl17:0_16:1 (39%) in the IM (Figure 3B). 

The second largest PL class found in *L. gormanii* membranes was PC. The major molecular PC species were as follows: PC15:0_15:0 (22% in the OM and 20% in the IM), PC15:0_16:0 (19% in the OM, 22% in the IM); PCcyclopropyl17:0_16:0 (15% in the OM, 20% in the IM), and PC cyclopropyl 17:0/15:0 (10% in the OM and 9% in the IM). PC cyclopropyl17:0_15:0 was characteristic for this class (Figure 3C). The distribution of PC species in the membranes was very similar as indicated by the high value of Spearman’s rank correlation coefficient (ρ ≥ 0.90).

The PC and PE content in *L. gormanii* membranes changed significantly when the bacteria were cultured on a medium with the addition of exogenous choline. The PC content in the OM and IM increased by 25% and 28%, respectively, compared to the bacteria cultured without choline supplementation (Figure 2). The amount of PE contained in the OM was by 18% lower than in *L. gormanii* cultured without exogenous choline. The content of PE in the IM was 32% lower than in the bacteria grown without choline addition (Figure 2). The comparison of the molecular profile of PC species contained in the membrane lipids of *L. gormanii* cultured on the medium with and without choline showed quantitative differences in the PC pool (Figure 3C). The content of PC cyclopropyl17:0_15:0 in the IM lipids isolated from bacteria grown on the medium with exogenous choline was approx. twice as high as in the IM lipids from bacteria grown without choline. Also, PC cyclopropyl17:0_16:0 was present in the IM in higher quantities compared to the IM lipids isolated from *L. gormanii* cultured without choline. The OM lipids from the bacteria grown on the choline-supplemented medium contained more PC15:0_15:0 than the bacteria grown on the non-supplemented medium. Quantitative differences in PE species between bacterial membranes from different growth conditions were found (Figure 3A). The content of PE15:0_15:0, PE15:0_16:0, and PE16:0_16:1 was higher in IM lipids extracted from the bacteria cultured with choline addition. There were no significant differences in PE species present in the OM lipids from both conditions. The bacteria cultured on the medium with exogenous choline contained mainly dMePE cyclopropyl17:0_16:1, dMePE16:1_18:1, and dMePE cyclopropyl17:0_16:0 in the OM and dMePE cyclopropyl17:0_16:1 and dMePE16:1_18:1 in the IM. Compared to the bacteria grown on the medium without the addition of choline, they contained less dMePE cyclopropyl17:0_17:0, dMePE16:0_16:1 and more dMePEcyclopropyl17:0_16:1 in the OM (Figure 3B). There were clear differences in the content of dMePE in the OM of bacteria grown on the medium with and without the addition of choline, as indicated by the negative Spearman correlation coefficient (−0.49).

*L. gormanii* lipids contained also PG, the amount of which in the OM was higher than in the IM and did not change depending on the growth conditions. Twenty-two PG species were identified in both membranes. The major molecular species were composed of 15:0 and 16:0, similar to the PE and PC classes (Figure 3D). However, unlike the PE and PC classes, PG16:1_16:1 was abundant in the OM lipids. The OM lipids contained more PG15:0_15:0, PG16:1_14:0, and PG15:0_14:0 than in the IM, while the IM lipids were characterized by higher amounts of PG15:0_16:0, PG15:0_17:0, and PG16:1_17:0 compared to the OM. 

The profile of CLs showed that CL66:1 and CL64:2 were the most abundant species. These species were found in both membranes. CL64:2 in the IM of the bacteria grown on the standard medium represented about 8%, while the IM of the bacteria grown on the choline-supplemented medium accounted for 45%. The content of this cardiolipin in the IM was about 14%, regardless of the growth conditions. CL66:1 was dominant in the IM of the bacteria cultured on the standard medium and accounted for 87% of all cardiolipins. In the OM, it represented 57%. The bacteria grown with the addition of exogenous choline synthesized lower amounts of this cardiolipin in both the OM and IM (44% and 23%, respectively).

### 2.5. Analysis of Glycerolipids

**Figure 4:**
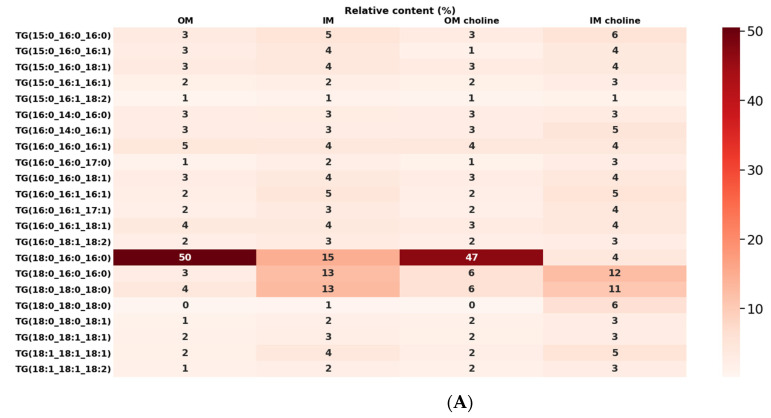

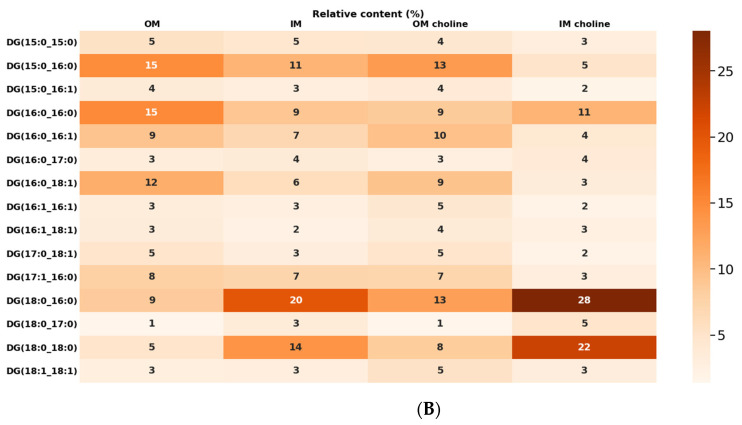
Profile of TG (**A**) and DG (**B**) of the OM and IM of *L. gormanii* grown on the medium without and with exogenous choline. Lipids were identified via LC-MS(/MS) in the positive ionization mode.

Glycerolipids (TG and DG) were the second most abundant fraction of lipids from *L. gormanii* membranes. TG constituted the most diverse class of lipids including 125 species dominated by TG18:0_16:0_16:0. This lipid comprised approx. 50% of total TG species and was mainly localized in the OM. The other TG species present in significantly smaller amounts were as follows: TG16:0_16:1_18:1, TG15:0_16:0_16:0, TG16:0_16:0_16:1, TG15:0_16:0_18:1, and TG18:0_18:1_18:1. The content of TG18:0_18:0_18:0 was 4% in the OM, and 13% in the IM (Figure 4A). Fatty acids with a hydrocarbon chain length of 16 and 18 were the main acids building of the lipid class. There were no significant differences in the content of TG species between the different growth conditions except for TG18:0_16:0_16:0, which was more than 3 times higher in the IM than the IM choline. However, the amount of TG 18:0_18:0_18:0 was higher in IM compared to IM choline.

In total, 27 DG molecular species were identified in the lipids of the *L. gormanii* membranes (Figure 4B). They were characterized by lower heterogeneity than TGs. Two DG species were dominant in the IM: DG18:0_16:0 (20%) and DG18:0_18:0 (14%). In turn, DG15:0_16:0 (15%) and DG16:0_16:0 (15%) were the most abundant in the OM. The analysis of the molecular profile of DGs showed that many of these species contained 16 or 18 carbon acid residues, similar to TG species. *L. gormanii* grown on a medium with the addition of exogenous choline synthesized more DG18:0_16: 0 (28%) and DG18: 0_18:0 (22%) in the IM, while less DG16:0_16:0 (9%) in the OM compared to the bacteria cultured without exogenous choline.

### 2.6. Characteristic of Ceramides

**Figure 5:**
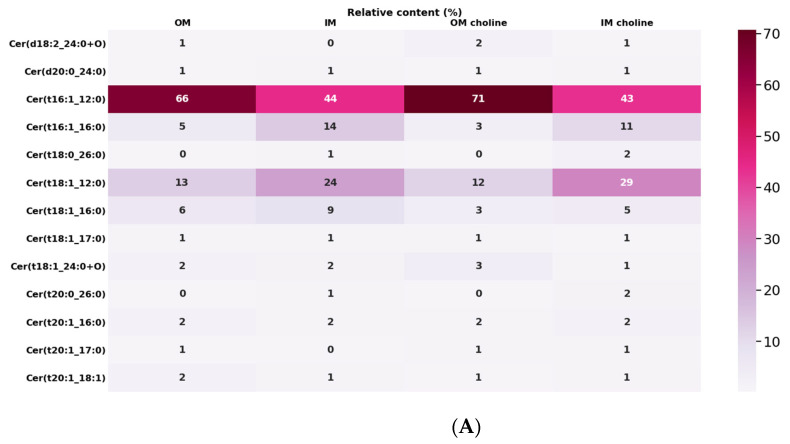

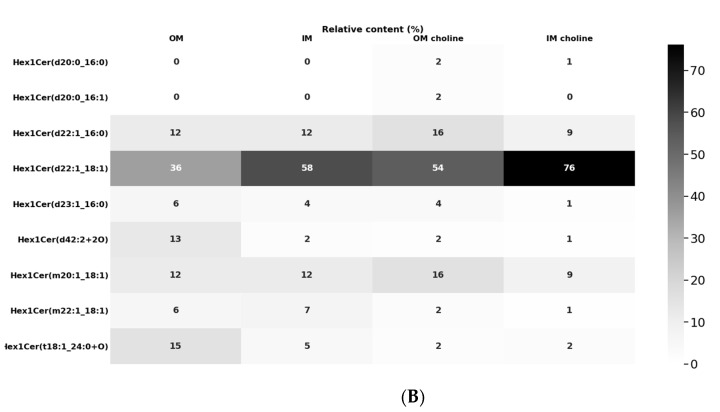
Ceramides (**A**) and hexosylceramides (**B**) in total lipid extracts of the OM and IM of *L. gormanii* cultured on the choline-supplemented and non-supplemented medium. Ceramides were analyzed by LC-MS(/MS) in the positive ionization mode.

Ceramides are a subtype of sphingolipids characterized by a long chain amino alcohol sphingoid backbone (in contrast to glycerolipids which have a glycerol backbone) with amide bound fatty acyl chains. The structural diversity of these lipids is related to differences in the length, degree of saturation, and methylation of fatty acids, as well as the variation in the lipid headgroup. *L. gormanii* synthesizes 23 various ceramide species with saturated and one-unsaturated FAs with a 12 to 24, as well as oxidized ceramides and more complex hexosylceramides (Hex1Cer) containing sugar moieties (Figure 5A,B).

**Figure 6:**
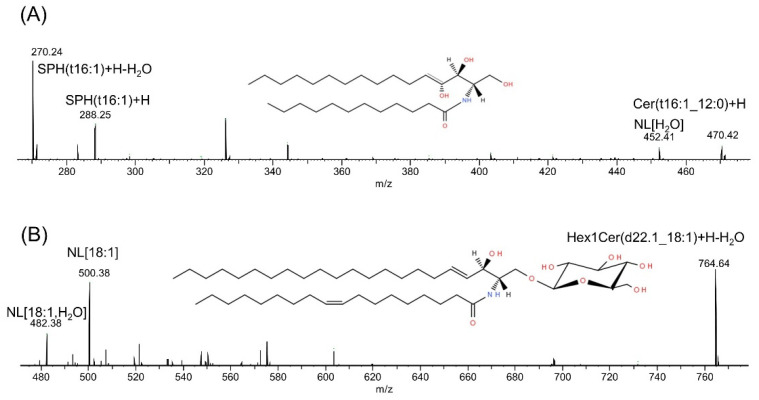
Fragmentation mass spectra in the positive ion mode of Cer(t16:1_12:0) (**A**) and Hex1Cer(d22:1_18:1) (**B**) Abbreviations: NL neutral loss, SPH sphingosine. The position of double bond was not identified in 16:1, 22:1 and 18:1 fatty acids.

In Figure 6A,B, the fragmentation mass spectra of the most dominant ceramide and hexosylceramide of *L. gormanii* are shown. 

All ceramides were distributed in the OM and IM, but there were clear quantitative differences for some species between the membranes. Among the total 14 ceramides and oxidized ceramides, one species, cer(16:1_12:0), represented approx. 66% of ceramides in the OM and approx. 44% in the IM in the case of the bacteria cultured without exogenous choline. The content of the cer(t16:1_12:0) (71%) species was higher in the OM, but similar in the IM of bacteria cultured with the addition of choline. Cer(t18:1_12:0) was the second most abundant species, which was localized mainly in the IM (24% or 29% IM choline), while its content in the OM was approx. half, regardless of the bacterial culture conditions (Figure 5A).

*L. gormanii* synthesizes three main hexosylceramides. These neutral glycosphingolipids, commonly called cerebrosides, usually contain glucose or galactose, with β-glycosidic linkages to the primary alcohol of an N-acyl sphingoid base. The dominant Hex1Cer22:1/18:1, accounting for 76% of total hexosylceramide species, was contained in the lipids isolated from the IM of the bacteria growing on the choline-supplemented medium. The content of this lipid in the OM was lower and amounted to 54%. The bacteria grown on a medium without choline supplementation compared to those supplemented with choline had a lower content of this lipid, i.e., 58% and 36% in the IM and OM, respectively (Figure 5B).

### 2.7. Bioinformatic Search for Ceramide Synthesis Pathways in the Genome of L. gormanii

**Figure 7:**
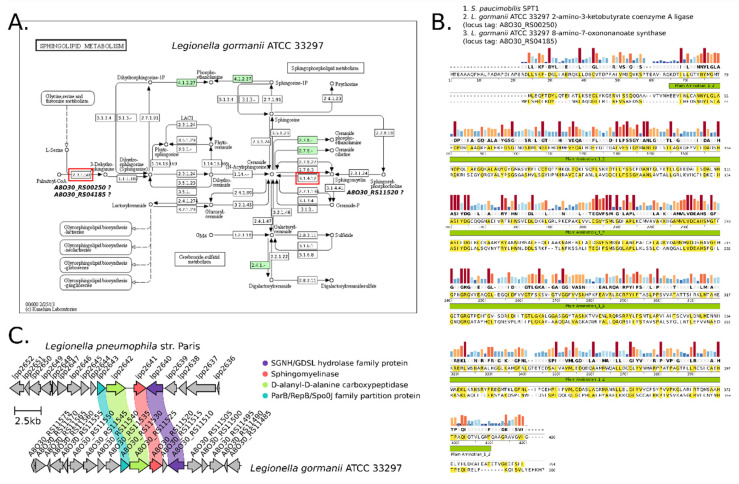
Searching for pathways of sphingolipid metabolism in the genome of *L. gormanii* ATCC 33297. (**A**) Mapping the *L. gormanii* ATCC 33297 genome annotation to the KEGG sphingolipid metabolism pathway map (map00600). Chemical compounds are represented as circles, and gene products are represented as rectangles. The two important entry enzymes in the pathway: serine palmitoyltransferase (SPT, EC 2.3.1.50, K00654) and sphingomyelin phosphodiesterase (SMPD1, 3.1.4.12, K12350) are outlined in red. Green rectangles represent enzymes found in the genome of *L. gormanii* ATCC 33297 via KofamKOALA and with assigned KO identifiers. For the first step of sphingolipid synthesis, i.e., condensation between serine and the acyl-CoA thioester, no SPT enzyme was found in the *L. gormanii* ATCC 33297 genome, based on the standard thresholds for KO identification. However, there are sequences encoding SPT protein homologues (8-amino-7-oxononanoate synthase, locus tag: A8O30_RS00250; 2-amino-3-oxobutyrate coenzyme A ligase, locus tag: A8O30_RS04185) present, belonging to the bacterial α-oxoamine synthases family. In the case of SMPD1, which catalyzes the breakdown of sphingomyelin into ceramide and phosphatidylcholine, although KO identification failed to assign this function, the homologue of predicted sphingomyelinase encoded by the lpp2641 *L. pneumophila* str. Paris gene with a level of identity of 61% was found in the *L. gormanii* ATCC 33297 genome. (**B**) Multiple protein sequence alignment of reference bacterial SPT from *Sphingomonas paucimobilis* (Q93UV0_SPHPI) and SPT homologues present in the genome of *L. gormanii* ATCC 33297. The color-coded bar at the top indicates the conservation degree. Below, there is the consensus sequence. In addition, the recognized Pfam domain (Pfam_Aminotran_1_2) is marked with a green rectangle, which indicates the presence of certain mechanistic features, such as the covalent binding of the pyridoxal-phosphate group to a lysine residue. The level of identity between *Sphingomonas paucimobilis* SPT and *L. gormanii* ATCC 33297 homologues is at the level of 30%. The alignment was generated using the Multiple Sequence Comparison by Log-Expectation (MUSCLE) algorithm in SnapGene (GSL Biotech). (**C**) Comparison of genomic regions encoding SMPD1 between in *L. pneumophila* str. Paris and *L. gormanii* ATCC33297. The individual coding sequences are marked with arrows according to their orientation. Above is the locus tag for the individual coding sequence in the GenBank database. The color lines joining the two regions indicate the level of identity above the designated threshold (0.22) for specific proteins. There are four CDSs with significant similarity within the analyzed regions coding for SGNH/GDSL hydrolase (violet), sphingomyelinase (red), D-alanyl-D-alanine carboxypeptidase (green), and ParB partition protein (green). This may indicate a common evolutionary origin of SMPD1 in these species. The comparison and visualization were made using the clinker tool.

To predict functional enzymes responsible for the synthesis of ceramides in *L. gormanii*, the genome of the strain ATCC 33279 was analyzed using the KEGG pipeline tools [13]. The reference point was a map describing the sphingolipid metabolism pathway (KEGG map00600), which includes the synthesis of ceramides. Enzymes with KO (KEGG Orthology) identifiers, found in the genome of *L. gormanii* ATCC 33279, were mapped into the discussed pathway (Figure 7A). According to the current knowledge, the first step in the synthesis of bacterial sphingolipids is the condensation of L-serine and palmitoyl-CoA to produce 3-ketodihydrosphingosine (KDS), a reaction catalyzed by the PLP-dependent enzyme serine palmitoyltransferase (SPT; EC 2.3.1.50, K00654) [14]. The coding sequences for SPT were not found in the genome, based on the standard KO number mapping pipeline. Additional searches with the use of PSI-BLAST and the sequence of the reference SPT from *Sphingomonas paucimobilis* (UniProt: Q93UV0_SPHPI) as a query allowed us to find two CDSs (GenBank locus tag: A8O30_RS00250 and A8O30_RS04185) in the genome that encode hypothetical proteins with the identity level of 31 and 25% to *S. paucimobilis* SPT respectively (Figure 7B). For a more complete picture, the annotation from NCBI (PGAP) [15] and PATRIC (RASTtk) [16] for these CDSs was checked and showed that CDS A8O30_RS00250 was annotated as “aminotransferase class I/II-fold pyridoxal phosphate-dependent enzyme” and “2-amino-3-ketobutyrate coenzyme A ligase (EC:2.3.1.29)”, while A8O30_RS04185 was annotated as “glycine C-acetyltransferase” and “8-amino-7-oxononanoate synthase (EC:2.3.1.47)”. Both 2-amino-3-ketobutyrate CoA ligase and 8-amino-7-oxononanoate synthase belong to the same protein family as SPT, the α-oxoamine synthase (AOS) subfamily of the larger group of pyridoxal-5′-phosphate (PLP)-dependent enzymes [17]. MSA of protein sequences of *S. paucimobilis* SPT and predicted proteins of *L. gormanii* A8O30_RS00250 and A8O30_RS04185 clearly shows conserved regions within the analyzed proteins (Figure 7B).

Ceramides can be synthesized de novo from serine and palmitate or produced by the cleavage of sphingomyelins by sphingomyelinases: SMase Cs (SMPD1, EC 3.1.4.12, K12350), which hydrolyze the ester bond between Cer and phosphorylcholine, and SMase Ds (EC3.1.4.41), which hydrolyze the phosphodiester bond between Cer-1-phosphate and choline [17]. Standard KO mapping gave negative results concerning the presence of the SMPD1 coding sequence in the *L. gormanii* genome (Figure 7A). However, in contrast to SPT, genes encoding for SMPD1 have already been found in other *Legionella* species. In particular, the lpp2641 gene in *L. pneumophila* strain Paris and the llo2622, llo1999, and llo1141 genes in *L. longbeachae* strain NSW150 were postulated to code for SMPD1 enzyme [17]. Using the lpp2641 translated sequence as a query in the blastp search against the *L. gormanii* genome, we found CDS with 61% identity, which can be regarded as coding for sphingomyelinase (A8O30_RS11520). To support this hypothesis, a comparison of the genomic regions surrounding the gene encoding SMDP1 in *L. pneumophila* str. Paris and *L. gormanii* was performed. In the analysis carried out done with the use of the clinker tool [18], a small synteny block is visible covering four genes for ParB partition protein, D-alanyl-D-alanine carboxypeptidase, sphingomyelinase and SGNH/GDSL hydrolase family protein, respectively (Figure 7C). This suggests a common evolutionary history of this gene block and makes the assumptions about the aforementioned CDS function plausible.

## 3. Discussion

Although bacteria and eukaryotic cells have evolved the ability to produce membranes with different compositions, *L. gormanii* synthesizes phosphatidylcholine and ceramides, i.e., typical components of eukaryotic membranes. Phospholipids were the predominant lipids in the OM and IM of *L. gormanii* cells. PE was the major class phospholipid in the *L. gormanii* membranes. The distribution of PE species in the membranes was similar except for PE15:0/16:0, where content in the IM was twice as high as in the OM, and PE cyclopropyl 17:0/17:0 with an amount in the OM was twice as high as in the IM.

The presence of methylated PE derivatives in the *L. gormanii* membranes indicates that one of the ways of PC synthesis in these bacteria is a pathway based on the triple methylation of PE. The pathway is catalysed by one or more phospholipid *N*-methyltransferases (PmtA) in various organisms [19]. The *Legionella* PmtA enzymes, encoded by *pmtA* genes, exhibit homology to the *Rhodobacter* Pmt-type enzyme, which in turn is homologous to UbiE (ubiquinone/menaquinone biosynthesis methyltransferase). However, in the previous studies, it was not possible to obtain a sequence of the *L. gormanii* pmtA gene using several degenerative primer pairs homologous to *Legionella*, and *Rhodobacter* bacteria [10]. Similar results were obtained by mapping the complete genome sequence of *L. gormanii* ATCC 33297 to the glycerophospholipid metabolism pathway (KEGG map 00564). Neither phosphatidylethanolamine N-methyltransferase (EC 2.1.1.17) nor phosphatidyl-N-methylethanolamine N-methyltransferase (EC 2.1.1.71) coding sequences have been identified in the genome of this strain. The lack of the *pmtA* homologue in *L. gormanii* genome, like *L. dumoffii*, may indicate the presence of a new type of enzyme with phospholipid *N*-methyltransferase activity in these blue–white fluorescent *Legionella* [10]. PC is the second most abundant *L. gormanii* phospholipid distributed in a similar amount in the OM and the IM. The molecular PC profile in both membranes was similar, but there were quantitative differences in the PC species. The IM contained higher content of PCcyclopropyl17:0/17:0 and PCcyclopropyl17:0/16:0, and lower amounts of PC15:0/16:1 than the OM. *L. gormanii* synthesizes PC also in a one-step process of direct choline condensation with CDP-diacylglyceride [10]. The bacteria grown with the addition of exogenous choline synthesized by 10% and 20% higher levels of PC in the OM and the IM, respectively, than the bacteria grown on the standard medium. The comparison of the pattern of PC species synthesized in the PmtA and Pcs pathways showed no differences in the PC pool originating from both pathways. However, *L. gormanii* grown on the choline-supplemented medium synthesized more PC species with cyclopropane acid (e.g., cyclopropyl17:0/15:0, cyclopropyl17:0/16:0) than the bacteria grown without the addition of choline. A characteristic feature of all classes of *L. gormanii* PLs was the high content of cyclopropyl17: 0 fatty acid. The presence of the acid with a cyclopropane ring, which is more stable than the double bond may increase the stability of *L. gormanii* membranes and thus be essential for the survival of the bacteria in the environment. Further analyses are needed to conclude its significance in environmental persistence. 

The presence of ceramides is an unusual feature of *L. gormanii* membrane lipids. Sphingolipids (SphL) are important components of the plasma membrane of mammalian cells, where they are typically localized in the outer leaflet [20]. They are also membrane components of a narrow group of bacteria mainly associated with a eukaryotic host. Most of the sphingolipid producing bacteria belonging to the Bacteroidetes phylum (e.g., such genera as *Flectobacillus*, *Porphyromonas*, *Prevotella*, *Parabacteroides*, *Bacteroides*) and the Chlorobi phylum (e.g., *Chlorobium*). Bacteria that synthesize sphingolipids also represent α-Proteobacteria (*Acetobacter*, *Sphingomonas*, *Novosphingobium*) and ∆-Proteobacteria (*Myxococcus*, *Bdellovibrio*) [20]. In bacteria, SphL seem to be localized in the outer leaflet of the outer membrane [21]. The ceramides of *L. gormanii* were found in both the OM and IM, but one species, i.e., cer(16:1_12:0), accounted for 50% of the ceramides present in the OM. The *N*-acyl groups of *L. gormanii* ceramides were even-chain length, long-chain saturated or monoenoic fatty acids with 16C, 18C and 20 to 24C. The structure of *L. gormanii* ceramides is similar to that of mammalian ceramides, whose acyl chain lengths range from 14 to 26 carbon atoms (or greater), with palmitic (C16:0) and stearic (C18:0) acids as the most common fatty acids. In contrast, fatty acids attached to the sphingoid backbones of bacterial sphingolipids are often odd-chain length, methylated, or hydroxylated [21]. *L. gormanii* also produces two species of oxidized ceramides and the ceramide with one hexosyl group. The synthesis of *L. gormanii* ceramides and their role, besides the structural function, remain to be clarified. The bioinformatic search for genes encoding proteins involved in the synthesis of sphingolipids showed that *L. gormanii* encodes hypothetical proteins with the level of identity of 31 and 25% to *S. paucimobilis* SPT respectively. 

Some pathogenic bacteria are able to degrade sphingolipids of the host cell and thus counteract the host cell’s response and promote intracellular replication. *L. pneumophila* encodes three enzymes: a sphingomyelinase, a sphingosine kinase and a sphingosine-1-phosphate lyase [22,23]. The activity of a sphingosine-1-phosphate lyase (LpSpl) prevents an increase in the level of sphingosine in macrophages infected with *L. pneumophila* and thus inhibits the process of autophagy [24]. *L. gormanii* encodes a hypothetical sphingomyelinase with a similarity of 61% to *L. pneumophila*.

## 4. Materials and Methods

### 4.1. Strain and Growth Condition

*Legionella gormanii* (ATCC 33297) strain was cultured at 37 °C for 96 h on buffered charcoal yeast extract (BCYE) agar plates (Oxoid, UK) or on this medium enriched with 100 μg/mL choline chloride (Sigma-Aldrich, St. Louis, MO, USA). Bacterial cells, harvested by centrifugation were washed twice in 0.5 M NaCl, once in distilled water, and lyophilised. The dry bacterial mass underwent the lipid extraction procedure.

### 4.2. Isolation of Phospholipids and Thin-Layer Chromatography

The Bligh and Dyer method (1959) with a chloroform–methanol–water mixture was used to extraction of lipids from 200 mg of the bacteria cultured on the medium without and with exogenous choline [25]. Two-dimensional thin-layer chromatography (TLC) on silica gel 60 F254 plates (Merck, Tokyo, Japan, 20 × 20 cm) was used to separate PLs into individual classes. The plates were washed twice with chloroform:methanol (1:1, *v*/*v*) and activated at 180 °C before use. PLs were spotted in one corner of the plate and subsequently separated using a solvent system consisting of chloroform, methanol and water (14:6:1, *v*/*v*/*v*) in the first dimension and chloroform, methanol and acetic acid (13:5:2, *v*/*v*/*v*) in the second dimension. Visualization of PLs was achieved using a charring plate with a solution of 5% sulfuric acid in methanol followed by heating to 180 °C for approximately 1 min. Identification of individual PLs was made by comparing R_f_ coefficients of individual components for both systems of solvents in comparison with R_f_ values for standard lipids. Synthetic phosphatidylcholine, phosphatidylethanolamine, phosphatidyl-*N*,*N*-dimethylethanolamine, phosphatidylglycerol, and cardiolipin from the bovine heart (Sigma-Aldrich, St. Louis, MO, USA) were used as standards.

PLs (1 mg) were applied about 1 cm from the bottom of the silica plate as a narrow band and separated with chloroform:methanol:acetic acid (13:5:2, *v*/*v*/*v*). The bands containing individual PLs were visualized with iodine vapour and next scraped off, transferred to screw-capped tubes, and extracted from silica gel with a mixture of chloroform:methanol (1:1, *v*/*v*). These separations were carried out in triplicate and the obtained fractions of individual phospholipids were intended for the analysis of the fatty acid composition. 

### 4.3. Preparation of Fatty Acid Methyl Esters 

Individual PL fractions were saponified in the presence of 1 mL of 0.8 M sodium hydroxide in 50% methanol on a heating block at 80 °C for 1 h. After cooling to room temperature, the samples were acidified with 6N HCl to pH 2 and then evaporated to dryness with an evaporator at 40 °C (Büchi, Rotavapor R-100, Postfach, Switzerland). Extraction of released fatty acids was carried out by 1.5 mL of a mixture of chloroform:water (1:2 *v*/*v*) and mixed by vortex for 2 min. The mixture was centrifuged at 5500× *g* at 10 min (Sigma, 6-KS). The upper aquatic phase was withdrawn with the aid of the Pasteur pipette and a new portion of water was added to the chloroform layer. After being shaken vigorously, the sample was centrifugated as above, and the organic phase was dried with anhydrous sodium sulphate and the chloroform was removed in the nitrogen stream. Next, 1 mL of 1M HCl/methanol (prepared from acetyl chloride) was added and the mixture was heated at 85 °C for 1.5 h to methylate free fatty acids. After evaporated to dryness with an evaporator, the fatty acid methyl esters (FAMEs) were extracted using 1 mL of a mixture of chloroform and water (1:2 *v*/*v*), centrifugated as above and the upper aquatic layer was discarded. The chloroform layer was additionally washed twice with 1mL of MQ water. Then the chloroform solution of FAMEs was dried with anhydrous sodium sulphate and the chloroform was removed in the nitrogen stream. FAMEs were resuspended in 50 µL chloroform before chromatographic analysis. 

### 4.4. Gas-Liquid Chromatography and Mass Spectrometry

The analysis of FAMEs was carried out using a gas chromatograph (Agilent Technologies, instrument 7890A) connected to a mass selective detector (Agilent Technologies MSD 5975C, inert XL EI/CI) (GLC-MS), using helium as a carrier gas. The chromatograph was equipped with an HP-5MS column (30 m × 0.25 mm). The temperature program was as follows: 150 °C for 5 min. raised to 310 °C (5 °C min^−1^), and the final temperature was maintained for 10 min.

FAMEs were identified by an analysis of their mass spectra and fragmentation patterns. Each fatty acid was quantified by calculating its peak area relative to the total peak area. The positions of the branching methyl group and the double bonds were determined by an analysis of mass spectra of fatty acid pyrrolidides [26]. The *cis* and *trans* isomers of 16:1 and 18:1 FAs were identified based on their retention times.

### 4.5. Separation of IM and OM in a Sucrose Density Gradient

*L. gormanii* cells harvested from 6 BCYE agar plates with or without exogenous choline were washed twice in cold 10 mM HEPES (N-2-hydroxyethylpiperazine N-2-ethanesulfonic acid; Sigma-Aldrich, Steinheim, Germany) buffer (pH 7.4) and centrifuged at 8000× *g*, 15 min at 4 °C (Sigma, 6-16KS). The cell pellets were suspended in 15 mL of 10 mM HEPES buffer containing 20% sucrose (*w*/*v*) and incubated with DNase (0.3 mg) (Sigma-Aldrich, Steinheim, Germany) and RNase (0.3 mg) (Sigma-Aldrich, Steinheim, Germany) at 37 °C for 30 min. The cells were lysed through a French Press (SLM-Amico Instruments, Thermo Spectronic, Rochester, NY, USA) three times at 18,000 psi and these lysates were centrifuged at 1000× *g* for 20 min at 4 °C. Total membrane fractions collected by centrifugation for 60 min at 100,000× *g* at 4 °C (SW 32Ti rotor, Beckman Coulter, Brea, CA, USA) and were washed twice in cold 10 mM HEPES buffer. The pelleted membranes were suspended in 2.5 mL of 10 mM HEPES buffer and layered onto a seven-step sucrose gradient consisting of 6 mL 70%, 9 mL 64%, 8 mL 58%, 5 mL 52%, 4 mL 48%, 3 mL 42%, and 3 mL 36% (*w*/*v*) in 10 mM HEPES buffer at pH 7.5. The samples were centrifuged at 114,000× *g*, 20 h, at 4 °C (SW 32Ti rotor, Beckman Coulter, Brea, CA, USA). The fractions were manually collected from the top of the gradient in 1-mL steps and were used to identify the IM via nicotinamide adenine dinucleotide (NADH) oxidase activity assay as described [27], and the OM by esterase activity as described previously [28]. Fractions containing the IM and the OM were combined separately. Next, the IM and OM were suspended in 10 mM HEPES buffer, centrifuged at 54,000× *g* for 1 h (MLA 80, Optima MAX-XP, Beckman Coulter, Brea, CA, USA), and washed twice in cold deionized water. The samples were suspended in MQ water and lyophilized. The concentration of protein in the fractions was determined using the Bradford method and bovine serum albumin as a standard [29]. Lipids isolated from the IM and OM according to the procedure described in Section 2 were subjected to liquid chromatography coupled to tandem mass spectrometry (LC-MS/MS). 

### 4.6. Ultra-High Performance Liquid Chromatography/Mass Spectrometry (UHP LC-MS/MS)

Ultrapure water, NH_4_formiate, isopropanol, chloroform, acetonitrile, and formic acid were obtained from Merck (Darmstadt, Germany) in LC MS grade purity.

The samples were transferred to glass vials and dried in a SpeedVac concentrator. Dried samples were reconstituted in 900 µL 20% of mobile phase B and 100 µL chloroform and centrifuged. A total of 3 µL was injected into the UHPLC/MS system. 

Lipids were then directly analyzed using a Vanquish UPLC-System (Thermo Scientific, Waltham, MA, USA) with a heated electrospray ionization (HESI) QExactive plus Orbitrap mass spectrometer (Thermo Scientific, Waltham, MA, USA). Chromatographic separation took place on a reversed-phase column (Accucore Polar Premium 100 × 2.1 mm (2.6 µ) with guard column: Accucore Polar Premium 10 × 2.1 mm (2.6 µ)) from Thermo Scientific (Waltham, MA, USA). Autosampler temperature was 10 °C throughout the whole measurements. 

Mobile phase A consisted of 60% acetonitrile, 10 mM NH_4_formiate and 0.1% HCOOH in ultrapure water. Mobile phase B was 90% isopropanol, 10 mM NH_4_formiate and 0.1% HCOOH in ultrapure water. Separation was performed with an increasing gradient of B (20–100% from 0.5 to 8.5 min and 20% from 12.5 to 15 min) over a total time of 15 min. Flow rate employed was 0.4 mL/min and the column temperature was 55 °C. The analytes were detected using a Thermo Orbitrap mass spectrometer equipped with a HESI source operated in the positive and negative ion mode. MS data were acquired over a scan range of 250–1200 *m*/*z* with full MS resolution of 70,000 and data-dependent MS² resolution of 17,500.

Identification and quantification of individual lipid species were performed by LipidSearch Software from Thermo Scientific (Waltham, MA, USA) on product level (MS/MS fragmentation) (Supplementary Materials).

### 4.7. Bioinformatic Analysis

#### 4.7.1. Sequence Retrieval and Gene Annotation

*L. pneumophila* str. Paris (GCA_000048645.1) and *L. gormanii* ATCC33297 (GCA_001648685.1) genomes were downloaded from National Center for Biotechnology Information (NCBI) as .gbff files, containing PGAP annotation [15]. For comparison and complement, the annotation obtained using RASTtk [16] from the PATRIC [30] website for both genomes were also downloaded. Protein sequence of SPT enzyme from *Sphingomonas paucimobilis* was downloaded from UniProtKB [31] with ID Q93UV0_SPHPI. 

#### 4.7.2. BLASTp and PSI-BLAST Searches

Homologs of SPT were searched using PSI-BLAST [32] with iterations and default thresholds against *L. gormanii* ATCC33297 genome with Q93UV0_SPHPI sequence as a query. Homologs of lpp2641 gene were searched by BLASTp against *L. gormanii* ATCC33297 genome also with default thresholds. 

#### 4.7.3. K Number Assignment and KEGG Mapping

All predicted protein coding sequences of *L. gormanii* ATCC33297 genome were subjected to K number assignment with the use of BlastKOALA and KofamKOALA tools [13,33]. After assignment, all proteins were linked to the KEGG pathways and EC numbers [33]. 

#### 4.7.4. Gene Cluster Comparisons

Genomic regions spanning approximately 10 kbp upstream and downstream from loci: lpp2641 in *L. pneumophila* str. Paris and A8O30_RS11520 in *L. gormanii* ATCC33297 were manually extracted using SnapGene software (GSL Biotech) and submitted to comparison using clinker tool with identity threshold set to 0.22 [18].

#### 4.7.5. Statistical Analysis

Results were statistically evaluated using Spearman’s rank correlation coefficient.

## 5. Conclusions

The in-depth analyses of the lipid components of the OM and IM in the *L. gormanii* cell wall showed that this bacterium contains components that are unique to prokaryotes but characteristic for eukaryotes. PC was the second most abundant PL after PE distributed in the OM and IM in a similar amount. The bacteria grown on a medium with exogenous choline increased the PC content in the OM and IM by 25 and 28%, respectively, and reduced the PE content in the OM and IM by 19 and 29%, respectively. The comparison of the molecular profile of PC species contained in the lipids of *L. gormanii* membranes cultured on the medium with and without exogenous choline showed only quantitative differences in the PC pool. *L. gormanii* synthesized ceramides with only an even number of carbon atoms in the acyl chains, which makes them more similar to mammalian than bacterial ceramides. These compounds were found in both the OM and IM but one species, i.e., cer(16:1_12:0), accounted for 65% of the ceramides present in the OM. The addition of exogenous choline to the growth medium increased the content of cer(16:1_12:0) in the OM to 70%.

As shown by the bioinformatic analyses of the *L. gormanii* genome, this bacterium encodes putative enzymes that may be involved in both de novo ceramide synthesis and sphingolipid degradation.

The presence of such macromolecules as PC and ceramides in *L. gormanii* membranes may be an example of the molecular mimicry used by these bacteria to infect a eukaryotic host cell effectively. Investigation of the phenotypic plasticity of the surface structures of *Legionella* bacteria is essential for understanding the pathogenesis mechanisms behind the evolutionary success of these bacteria.

## Figures and Tables

**Table 1:** Fatty acid composition of individual classes of *L. gormanii* phospholipids identified by FAME analysis via GC/MS. Fatty acids were liberated by saponification (0.8 M NaOH/50% methanol, 1 h, 80 °C) and esterified by 1 M HCl/methanol (1.5 h, 85 °C).

Relative Content [%]						
Retention Time	Fatty Acid	PE + mMePE	dMePE	PC	PG	CL
9.67	*i*14:0	0.5 ± 0.2	0	2.5 ± 0.2	0	tr
10.49	*n*14:0	tr	1± 0.2	0.5 ± 0.2	1 ± 0.4	1 ± 0.2
11.81	*i*15:0	tr	0	tr	0.5 ± 0.2	tr
11.97	*a*15:0	**19 ± 2**	1 ± 0.3	**8 ± 2**	**17 ± 2**	**10 ± 0.5**
12.33	9 *-15:1	0	0	0	0	0.6 ± 0.2
12.62	*n*15:0	3 ± 0.3	1 ± 0.5	2 ± 0.2	2 ± 0.4	2 ± 0.4
13.90	*i*16:0	**22 ± 0.5**	1 ± 0.2	**12 ± 1**	**19 ± 1**	**15 ± 4**
14.25	*cis* 9 *-16:1	1 ± 0.2	1 ± 0.3	0.7 ± 0.1	1 ± 0.1	6 ± 0.7
14.43	*trans* 9 *-16:1	2 ± 0.4	0	1 ± 0.2	1 ± 0.6	0.5 ± 0.2
14.66	*n*16:0	**17 ± 0.4**	**25 ± 3**	**18 ± 2**	**17 ± 1**	**14 ± 2**
15.92	*i*17:0	1 ± 0.1	0	1± 0.2	2 ± 0.4	1 ± 0.2
16.09	*a*17:0	**7 ± 0.5**	**3 ± 0.5**	**5.5 ± 0.8**	**9 ± 0.1**	**7 ± 0.6**
16.467	*c*17:0	**18 ± 1**	**19 ± 2**	**22 ± 2**	**10 ± 1**	**15 ± 1**
16.673	*n*17:0	3 ± 0.3	2 ± 0.2	4 ± 0.5	3 ± 0.4	3 ± 0.2
18.531	*cis* 9 *-18:1	0	2 ± 0.5	0	tr	5 ± 0.6
18.810	*n*18:0	**6 ± 1**	**40 ± 1.5**	**16 ± 1.5**	**13 ± 0.3**	**14 ± 3**
19.690	*i*19:0	0	0	2 ± 0	1 ± 0.3	0.5 ± 0.1
20.362	*n*19:0	0.5 ± 0.1	0.5 ± 0.2	2 ± 0.4	2 ± 0.2	1 ± 0.2
20.584	*i*20:0	0	0	0	0	1 ± 0.3
21.675	20:1	0	0	0	0	1 ± 0.1
22.100	*n*20:0	tr	0	0.8 ± 0.2	0.7 ± 0.4	0.9 ± 0.2
23.740	21:0	tr	0	2 ± 0.8	0.8 ± 0.1	1 ± 0.2
25.346	22:0	0	1.5 ± 0.1	0	0	tr
28.303	24:0	0	2 ± 0.2	0	0	0.5 ± 0.1

*a*, methyl branch at the anteiso carbon atom; *i*, methyl branch at the iso carbon atom; *n*, unbranched acid; *c*, cyclopropane ring structure, tr—trace; * position of double bond. PE, phosphatidylethanolamine; mMePE, monomethylphosphatidylethanolamine; dMePE, dimethylphosphatidylethanolamine; PC, phosphatidylcholine; PG, phosphtidylglycerol; CL, cardiolipin.

## Data Availability

The data presented in this study are available in the article and Supplementary Materials.

## Supplementary Materials

The following supporting information can be downloaded at: https://www.mdpi.com/article/10.3390/metabo12050418/s1, Table S1: All lipid identifications showing accurate mass measurements at the MS and MS/MS level (identified lipid ions and corresponding productions in the positive ionization mode). Table S2: All lipid identifications showing accurate mass measurements at the MS and MS/MS level (identified lipid ions and corresponding productions in the negative ionization mode). Grade A: Class and all fatty acid chains are completely identified. Grade B: Both class specific ion and fatty-acid-derived product ions are detected. Grade C: Either class specific ion or fatty-acid-derived product ions are detected. Grade D: Unable to identify the lipid structure, such as a dehydrated ion.
